# Intensified ChlVPP/ABVVP chemotherapy regimen and pegfilgrastim support in advanced Hodgkin lymphoma

**DOI:** 10.3332/ecancer.2010.184

**Published:** 2010-09-08

**Authors:** E Cocorocchio, A Vanazzi, S Bassi, F Peccatori, P Antoniotti, F Gigli, L Travaini, G Piperno, G Pruneri, L Preda, R Biffi, E Botteri, M Negri, G Martinelli

**Affiliations:** 1 Haematoncology Division; 2 Pathology and Laboratory Medicine Division; 3 Nuclear Medicine Division; 4 Radiotherapy Division; 5 Radiology Division; 6 General Surgery Division; 7 Epidemiology and Biostatistics Division, European Institute of Oncology, Via Ripamonti, 435, 20141 Milan, Italy

**Keywords:** ChlVPP/ABVVP, intensified regimen, Hodgkin lymphoma

## Abstract

We present feasibility, toxicity and efficacy results of an intensified six-cycle ChlVPP/ABVVP regimen in advanced Hodgkin lymphoma (HL). From February 2004 to August 2007, 82 consecutive eligible patients were enrolled. According to the Hasenclever index, 64 patients (78%) were considered at low risk, 15 (18%) at intermediate and 3 (4%) at high risk. The most relevant toxicity was haematological: grade 3–4 neutropenia occurred in 32% of patients, grade 3–4 anaemia in 26% of patients. Severe infections and febrile neutropenia were observed in 8% of patients. With a median follow-up of 35 months (range 12–55), the three-year freedom from treatment failure (FFTF) and overall survival (OS) were 75% (95% CI 65%–86%) and 94% (95% CI 87%–99%), respectively. The intensified ChlVPP/ABVVP regimen in advanced HL is effective, does not seem to differ from standard regimens in terms of FFTF and OS and showed a favourable toxicity profile.

## Introduction

Adriamycin, bleomycin, vinblastine and dacarbazine (ABVD) chemotherapy (CT), in combination with radiotherapy (RT) when necessary, is considered the treatment of choice in advanced HL [1–4]. However, about one third of patients with complete remission (CR) will subsequently relapse [5,6]. In recent years, intensified CT schedules have been investigated and there are indications for an improvement in treatment results [7–10]. Nonetheless, long-term toxicities should be considered.

In 2003, Diehl *et al* [7] reported their experience with increased-dose BEACOPP, showing a possible benefit in terms of overall response rate (ORR), event-free survival (EFS) and overall survival (OS). More recently, Federico *et al* [10] published a randomised study comparing ABVD versus BEACOPP versus COPPEBVCAD-CEC: a better failure-free survival (FFS) and progression-free survival (PFS) with the BEACOPP regimen was found but without any advantage in OS and a high incidence of grade 3–4 toxicity.

In 2004, we reported our experience with the hybrid combination regimen ChlVPP/ABVVP in advanced HL [4]: cycles were repeated every 4 weeks, for a maximum of eight administrations. Clinical results demonstrated a relevant clinical activity in terms of ORR (96%), 5-year EFS (71%) and OS (79%). Considering that grade 3–4 neutropenia precluded the correct delivery of CT in 67% of patients, we decided to intensify the schedule introducing pegylated filgrastim (pegfilgrastim).

From February 2004 to August 2007, 82 consecutive patients were included, with the aim to investigate feasibility, toxicity and efficacy of an intensified ChlVPP/ABVVP regimen.

## Materials and methods

### Eligibility

All patients had histologically confirmed and newly diagnosed HL. Eligible patients had stage IIA with at least one of the following risk factors: bulky disease (defined as mediastinal mass ≥ 1/3 maximum transverse thorax diameter or any lesion ≥ 5 cm), extra-nodal involvement, erythrocyte sedimentation rate (ESR) ≥ 50, ≥ 3 lymph node regions involved or stage IIB, III and IV, with adequate renal and liver functions (serum creatinine level <2.5 mg/dl, total bilirubin level <1.5 mg/dl, AST/ALT level <2.5 times upper limit of normal). At time of treatment, absolute neutrophils count (ANC) should have been ≥1.5 x 10^9^/l, platelets (PLT) ≥ 150 x 10^9^/l and haemoglobin (HB) ≥ 9 g/dl.

Patients were excluded if they had received prior CT or RT, in case of any other malignancy or history of prior malignancy (except non-melanoma skin tumours or *in situ* cervical carcinoma), uncontrolled chronic disease, HIV infection, psychiatric illness or pregnancy.

The study was approved by the institutional review board, and written informed consent was obtained from all patients.

### Study design

Patients underwent full staging, including a full patient history and physical examination, computed tomography (CT) scans of the chest, abdomen and pelvis, ^18^F-fluorodeoxyglucose PET (^18^FDG-PET) scanning, complete blood count (CBC), ESR, a biochemical profile and a bone marrow trephine biopsy.

Treatment schedule was the following: day 1: vinblastine 6 mg/m^2^ intravenously; day 1 to 7: chlorambucil 6 mg/m^2^/d orally, procarbazine 80 mg/m^2^/d orally, prednisone 50 mg/d orally; day 8: doxorubicin 30 mg/m^2^, bleomicin 7.5 mg/ m^2^, vincristine 1 mg, intravenously; day 8 to 10: etoposide 100 mg/ m^2^/d intravenously; day 11: Pegfilgrastim was administered at the dose of 6 mg subcutaneous on day 11. Cycles were repeated every 21 days if ANC ≥ 1.0 x 10^9^/l and PLT ≥ 100 x 10^9^/l. On day 1 of each cycle, each patient had a physical examination, a CBC and blood biochemistry evaluation; on day 8 and 15 only a CBC. Antibiotic profilaxis was not given routinely. Consolidation RT was delivered to the site of bulky disease in patients with a CR.

**Figure 1:**
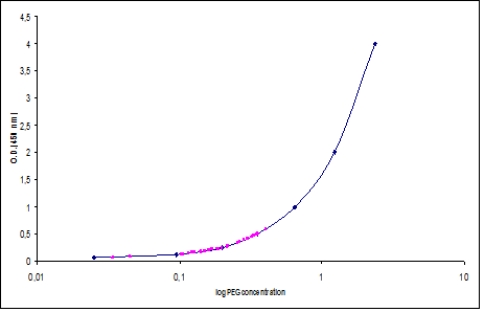
Pegfilgrastin serum concentration in 34 samples evaluated on day 1 of the cycle.

As the period between pegfilgrastim administration and the subsequent CT cycle was <14 days, to establish the optimal interval, an evaluation of pegfilgrastim serum concentration was performed for the first group of 11 patients [11]. On the first day of each cycle, the pegfilgrastim serum concentration was evaluated using Quantikine® (human G-CSF Immunoassay), an enzyme-linked immunosorbent assay (ELISA test) specific for filgrastim. A standard curve was prepared with an upper concentration of pegfilgrastim of 4 ng/ml and a lower concentration of 0.062 ng/ml (Figure 1). Pegfilgrastim concentration was reported on *x*-axis and optical density on *y*-axis. Sample concentrations were calculated by measuring the optical density of the serum and extrapolating from the standard curve, using PC software connected to the spectrophotometer.

Primary end points were the safety profile of the schedule in terms of toxicity, treatment intervals between cycles, evaluation of relative dose-intensity (RDI)—defined as the rapport between the administered dose/m^2^/week and the expected dose/m^2^/week. Secondary end points were FFTF and OS.

### Toxicity assessment

Adverse events were assessed according to the Common Toxicities Criteria of the National Cancer Institute (NCI, version 3.0) [12].

### Response assessment

Response evaluation was performed after three and six cycles of CT by a physical examination, CT scan of the neck, chest, abdomen and pelvis and an ^18^FDG-PET. A bone marrow biopsy was performed at the end of the CT program if positive for disease localization at baseline.

During follow-up, clinical evaluation by physical examination and biochemistry was performed every three months for up to two years, than every six months for up to five years, and thereafter annually until disease progression or death. Radiological evaluation was performed by CT scan every six months for up to five years, annually thereafter for up to 10 years.

### Statistics

The FFTF was calculated from the date of the first cycle to the date of disease progression after CR, or to the date of last follow-up in the case of prolonged CR. If a patient failed to achieve a CR, FFTF was set to zero. OS was defined as the time interval from the date of first cycle to the date of death or to the date of last follow-up if death did not occur. Survival curves were plotted using the Kaplan–Meier method. The log-rank test was used to assess survival differences between groups. The Kruskal–Wallis test was used to compare RDI medians. All analyses were carried out with the SAS software (SAS Institute, Cary, NC). All tests were two sided.

## Results

### Patient characteristics

From February 2004 to August 2007, 82 consecutive patients were included in the analysis. Their characteristics are listed in Table 1. Median age was 34 years (range 15–68 years), 42 out of 82 were male. Histology included 77 classical and 5 lymphocyte predominant HL (94% and 6%, respectively). According to the Hasenclever index [13], 64 out of 82 patients (78%) were considered at low risk, 15 (18%) at intermediate and 3 (4%) at high risk.

All patients received six cycles of CT. Involved field RT was performed in 26 patients, with bulky disease at diagnosis and with CR, with a median dose of 35.5 Gy (range: 25–37 Gy).

### Toxicity

Haematological and non-haematological toxicities are reported in Table 2.

76 out of 82 patients were evaluated for toxicity: grade 3–4 neutropenia occurred in 24 out of 76 patients (32%), grade 3–4 anaemia in 20 patients (26%). Transfusional support was required in 23 patients with a median of 2 (range 2–8) red cell units per patient. After the first 14 patients, darbopoietin (500 mcg sc q 3 weeks) was introduced when the haemoglobin level fell to <10 g/dl, reducing the rate of transfused patients from 57% to 24%. Febrile neutropenia and grade 3–4 infections were observed in six patients (8%).

Two patients experienced asymptomatic pulmonary embolism after the third and fourth cycle, respectively, while one patient developed a cavernous sinus thrombosis after the second cycle.

All three had received darbopoietin while in two of them, oral contraceptives given to preserve fertility were probably contributory.

10 patients (12%) delayed treatment (nine patients one cycle, one patient two cycles) with a median delay time of one week (range one—six weeks). Delays were due to symptomatic neurotoxicity (one case), infectious status (seven cases; varicella zoster infection, flu syndrome, pneumonia and febrile neutropenia), thrombocytopenia (one case), gastrointestinal disorder (one case), cavernous sinus thrombosis (one case).

A dose reduction of at least 20% of one drug occurred in 31 patients: 13 patients reduced the dose for 1 drug, 12 patients for 2 drugs and 6 patients for > 2 drugs of the schedule. Dose reduction was performed starting from the second cycle in eight patients, from the third cycle in eight patients, from the fourth and fifth cycle in the remaining 15 patients. The planned dose of doxorubicin and etoposide was reduced in three and nine patients, respectively; three patients reduced both, due to previous haematological or gastrointestinal toxicity. Vinca alkaloids dose reduction was mainly due to neurological toxicity. The RDI for each drug is reported in Table 3. The median RDI for all drugs was 96.95%. When these data were compared with those obtained from the previous experience with standard ChlVPP/ABVVP [4], the RDI resulted improved mainly for myelotoxic drugs, such as doxorubicin and etoposide.

### Pegfilgrastim

Pegfilgrastim serum concentration was evaluated in 34 samples (11 patients). The median level of pegfilgrastim serum concentration at day 11 was 0.27 ng/ml (range 0.07–0.59 ng/ml), well below the lowest filgrastim serum level concentration that can stimulate granulopoiesis (2 ng/ml), according to the biomathematical model that describes the concentration–effect relationship by an E-function [11] (Figure 1).

### Outcome

All patients were evaluable for response with an ORR of 99%. At the end of treatment, 73 out of 82 patients obtained a CR (89%), 8 patients a partial remission (PR) (10%) with histological confirmation of disease persistence available for 1, and 1 patient a progressive disease (PD) (1%).

Ten out of 73 CRs relapsed, with a median time to relapse of 9 months (range 4–38). Autologous stem cell transplantation (ASCT) was performed in 17 out of 19 failures, achieving a second CR in 12 patients. The remaining five patients underwent allogenic stem cell transplantion (AlloSCT), obtaining a CR in one case. ASCT was not performed in two patients because of elderly age in the first and sub-optimal performance status and rapid disease progression in the latter.

Five out of 82 patients died: four due to PD, one due to toxicity related to salvage CT. With a median follow-up of 35 months (range: 12–55) FFTF, and OS at 3 years were 75% (95% CI 65%–86%) (Figure 2a), and 94% (95% CI 87%–99%) (Figure 2b), respectively. When patient outcome was compared to risk profile, the low-risk group had a statistically significant advantage in terms of FFTF and OS compared to the intermediate risk group (p<0.001 and 0.002) (Figures 3a and b).

## Discussion

Our experience confirmed the safety and feasibility of the intensified ChlVPP/ABVVP regimen. A single injection of pegfilgrastim allowed optimal drug delivery, adequate dose intensity and a reduction in overall treatment duration if compared with our previously published four-week schedule [4].

The most relevant toxicity was haematological with grade 3–4 neutropenia in 32% of patients. Nonetheless, neutrophil recovery and the low rate of grade 3–4 infections make this schedule more favourable in terms of toxicity profile when compared to the standard ChlVPP/ABVVP or the standard/increased BEACOPP (Table 4). Symptomatic anaemia requiring transfusional support was one of the most relevant toxicities observed in the first 14 patients. Darbopoietin effectively reduced transfusional support, demonstrating the efficacy of this strategy [14]. However, thromboembolic events occurred in three patients: all had received darbopoietin, two patients also combined with oral contraceptives. It is well known that the use of darbopoietin and hormonal contraception are associated with a 1.5-fold and a 2.0-fold increased risk of thrombosis, respectively [15,16]. The concomitant administration of CT, darbopoietin and oral contraceptives should be reserved to selected cases and probably requires adequate prophylaxis.

In our previous experience [4], a four-week schedule without pegfilgrastim, haematological toxicity and consequent CT delays occurred in 67% of patients, influencing the RDI of the schedule. The use of pegfilgrastim allowed a RDI > 80% for each of the drugs included in the regimen. When compared to the ChlVPP/ABVVP regimen [4], the RDI was significantly increased for doxorubicn (90.7% vs 96%, p <0.01) and etoposide (68.6% vs 98%, p:<0.01) without increasing toxicity.

Second malignancies, MDS and secondary leukaemias represent the leading cause of excess mortality in Hodgkin disease survivors, correlated with the total CT dose and for RT with the total dose and fields involved [17–18]. The use of alkylating agents and etoposide is associated with an increased risk of short onset acute leukaemias, while solid tumours have emerged as the most significant late onset secondary malignancies [17–19]. The risk of solid tumours reported for patients who received sub-total or total nodal irradiation compared with involved field RT is 1.84-fold higher [17–19]. Although the median follow-up is still short, patients who underwent our intensified ChlVPP/ABVVP regimen did not develop MDS, secondary leukaemias or secondary solid tumours.

Clinical results in terms of FFTF (75%) and OS (94%) demonstrate the efficacy of the schedule. The use of ^18^FDG-PET in the routine management of HL patients introduced more stringent criteria for response evaluation [20], thus the CR rate in our previous experience (95%) [4] was probably overestimated. ^18^FDG-PET scan restaging at the end of treatment modified the management of these patients: ^18^FDG-PET positive patients underwent intensified CT with ASC support, while a negative ^18^FDG-PET identified patients who were probably cured by therapy. Relapse rate was different in the two groups (14% vs 24%), with an odds ratio of 2.0 (95% CI 0.8%–4.9%). This may explain the improvement in terms of OS in this cohort of patients versus the previous one (94% vs 79%).

Our results are slightly better than those reported for ABVD. When compared to standard BEACOPP [7], FFTF and OS are similar for patients with a low Hasenclever index, while the small number of patients with intermediate and high-risk index precludes any conclusion. Results of intensified BEACOPP seems to be better in terms of FFTF, also at 10 years, but not of OS [21]. The toxicity profile and late events make this schedule not always suitable for older patients or patients with co-morbidities.

The ASCT is an effective salvage programme [22], and an intensified ChlVPP/ABVVP regimen does not preclude stem cell mobilization since all patients taking part in the procedure successfully collected peripheral blood stem cells.

In conclusion, intensified ChlVPP/ABVVP demonstrated a favourable profile in terms of acute and long-term toxicity, and seems to be–-in terms of FFTF and OS—as effective as other standard regimens. Patients relapsing after intensified ChlVPP/ABVVP regimen can be cured with the ASCT.

## Figures and Tables

**Figure 2a:**
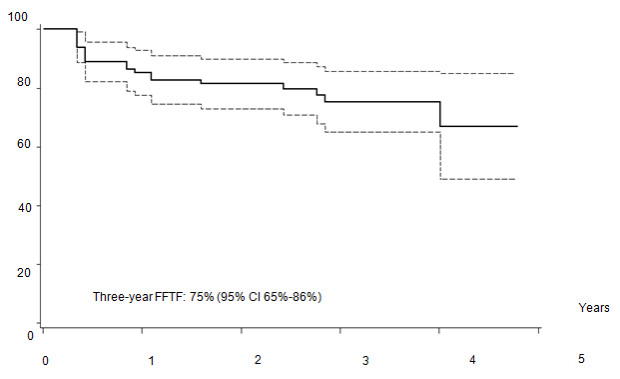
Freedom from treatment failure (FFTF).

**Figure 2b:**
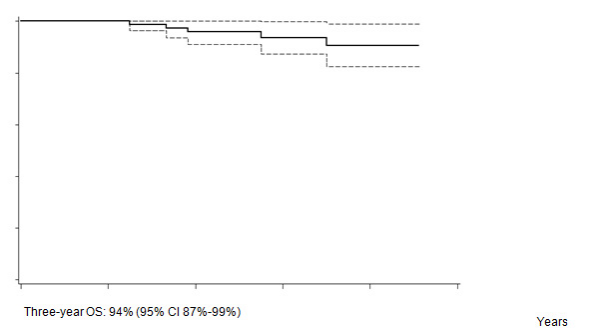
Overall survival (OS).

**Figure 3a:**
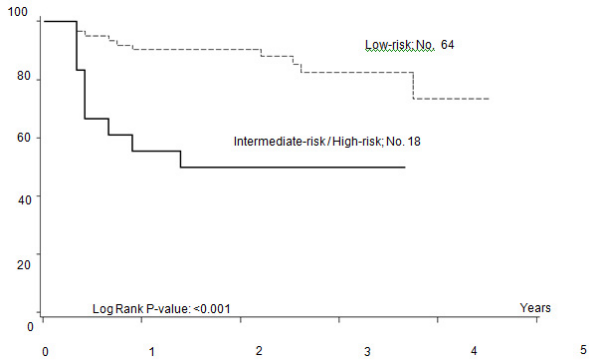
Freedom from treatment failure (FFTF) according to risk profile.

**Figure 3b:**
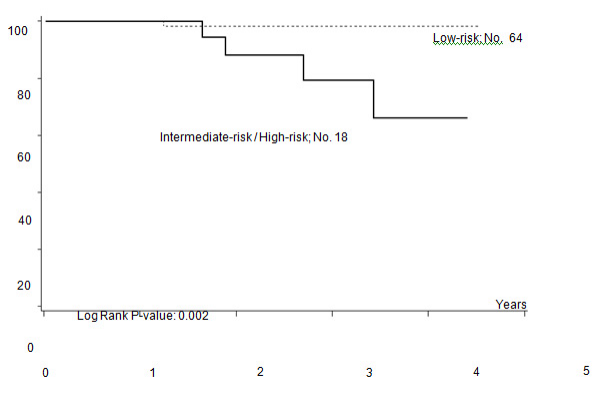
Overall survival according to risk profile.

**Table 1:**
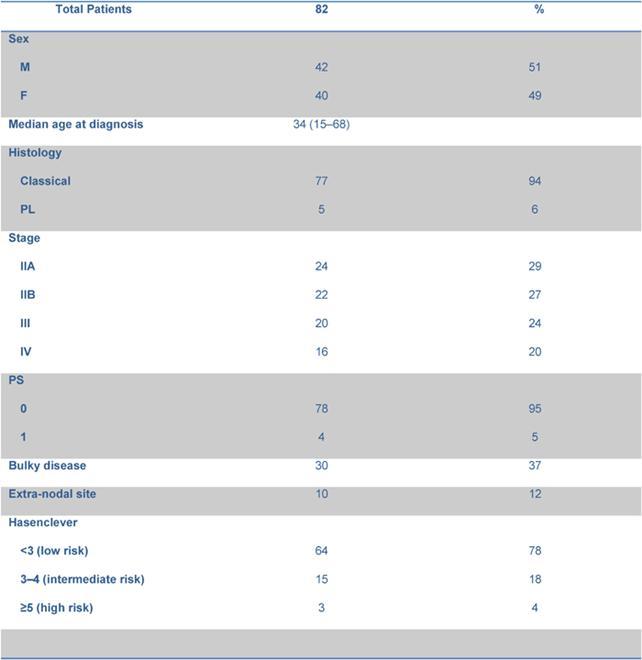
Patient characteristics and disease status.

**Table 2:**
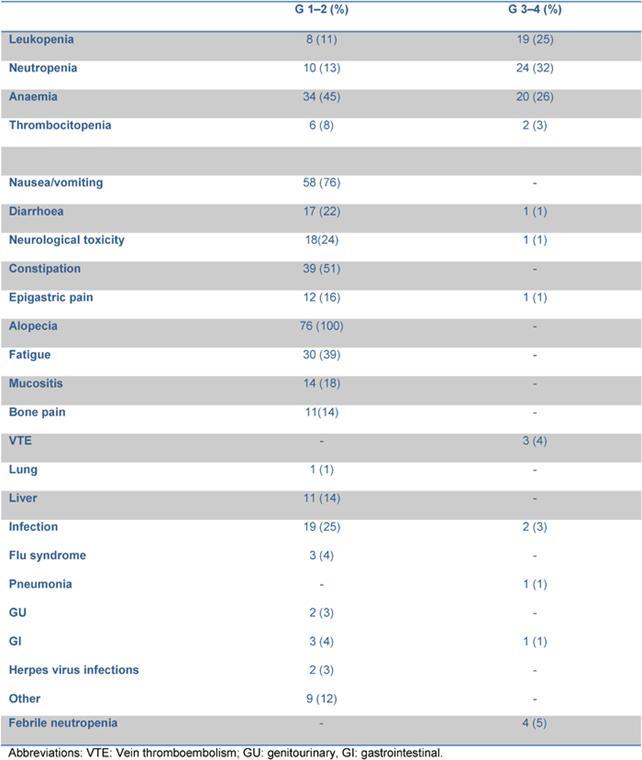
Haematological and non-haematological toxicity.

**Table 3:**
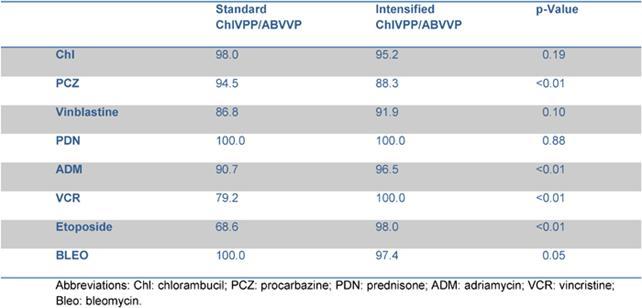
Comparison of relative dose intensity within standard and intensified ChlVPP/ABVVP.

**Table 4:**
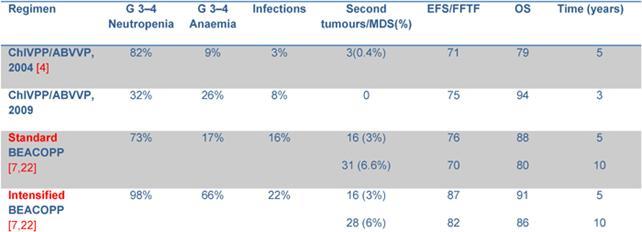
Grade 3–4 toxicity and outcome from other trials.
